# Non-Invasive Characterization of Locomotor and Ventilatory Responses in Rainbow Trout Under Acute Ammonia Nitrogen Stress

**DOI:** 10.3390/biology15131080

**Published:** 2026-07-06

**Authors:** Guanxu Li, Liu Yang, Ziyi Yin, Qihong Chen, Haoze He, Chengguo Wang

**Affiliations:** 1 Yantai Institute, China Agricultural University, Yantai 264670, China; liguanxu@cau.edu.cn (G.L.); yangliucau@cau.edu.cn (L.Y.); yinziyi@cau.edu.cn (Z.Y.); chenqihong@cau.edu.cn (Q.C.); hehaoze@cau.edu.cn (H.H.); 2 Shandong Key Laboratory of Digital Fishery, Yantai 264670, China

**Keywords:** ammonia nitrogen stress, rainbow trout, locomotor behavior, ventilation frequency, computer vision, non-invasive characterization

## Abstract

Ammonia nitrogen is one of the most common environmental stressors in aquaculture water environments, and its accumulation can induce physiological disturbance, altered ventilation regulation, and abnormal behavioral responses in fish. To achieve non-invasive quantitative characterization of rainbow trout responses to ammonia nitrogen stress, this study developed a computer-vision-based framework for the integrated analysis of locomotor behavior and ventilation activity. Rainbow trout were exposed to four ammonia nitrogen concentrations: 0, 15, 30, and 60 mg/L. A total of 16 rainbow trout were used in this study, with an average body length of 14.0 ± 1.0 cm and an average body weight of 38.65 ± 2.42 g. The fish were assigned to four experimental aquaria, with four fish maintained in one aquarium for each TAN treatment. Stereo videos for locomotor behavior analysis and monocular mouth-region videos for ventilation analysis were simultaneously collected, and the final 5 min of each recording was analyzed. YOLOv11n, multi-object tracking, and stereo vision were used to extract three-dimensional position sequences of rainbow trout and calculate the amount of exercise, average swimming speed, and spatial distribution. Meanwhile, optical-flow analysis was applied to quantify mouth opening–closing motion and estimate ventilation frequency. The results showed that with increasing ammonia nitrogen concentration, rainbow trout locomotor behavior tended to be suppressed, with average swimming speed showing the clearest decrease, whereas ventilation frequency continuously increased. Average swimming speed decreased from 3.83 cm/s in the 0 mg/L group to 1.03 cm/s in the 60 mg/L group, while ventilation frequency increased from 84.91 breaths/min to 133.43 breaths/min. Compared with locomotor indicators, ventilation frequency showed a more stable response to changes in ammonia nitrogen concentration. This study achieved the synchronous quantification of rainbow trout locomotor behavior and ventilation activity, revealing a differentiated response pattern characterized by enhanced ventilation and suppressed locomotor behavior under acute ammonia nitrogen stress. These findings provide a methodological reference for fish stress assessment and risk warning in aquaculture environments.

## Simple Summary

Ammonia nitrogen accumulation in aquaculture water can affect fish health and behavior, but many conventional assessment methods require manual observation or invasive measurements. In this study, we developed a non-invasive computer-vision-based method to quantify the responses of rainbow trout under acute ammonia nitrogen stress. Stereo video was used to analyze three-dimensional locomotor behavior, while monocular mouth-region video was used to estimate ventilation frequency from mouth opening and closing movements. The results showed that rainbow trout responded to increasing ammonia nitrogen concentration with enhanced ventilation and suppressed locomotor behavior. Ventilation frequency increased continuously, whereas average swimming speed decreased in the ammonia nitrogen stress groups. These findings suggest that combining locomotor and ventilatory indicators can provide a more comprehensive description of fish responses to ammonia nitrogen stress. The proposed visual analysis framework may help support fish stress assessment and aquaculture water-environment management.

## 1. Introduction

Aquatic products are an important source of high-quality protein, essential fatty acids, vitamins, and minerals for humans, and contribute substantially to global nutrition and dietary improvement [1]. According to the Food and Agriculture Organization of the United Nations [2], global aquatic food production continued to increase in 2025, highlighting the growing importance of aquatic products in global food supply and nutritional security. As the potential for further growth in capture fisheries becomes increasingly limited, aquaculture has emerged as a major driver of aquatic food production, playing an important role in ensuring nutritional security and supporting industrial development [3]. However, with the continued expansion of aquaculture and increasing environmental pressure, the deterioration of aquaculture water quality has become an increasingly prominent constraint. Water quality is now one of the key factors limiting the healthy growth of cultured animals and the sustainable development of aquaculture. Among the physicochemical factors affecting aquaculture water quality, ammonia nitrogen is one of the most common and important environmental stressors because of its diverse sources, high tendency to accumulate, and pronounced toxicity [4].

The decomposition of organic matter, including aquatic animal excreta and residual feed, increases the nitrogen load in aquaculture water and leads to elevated total ammonia nitrogen (TAN) concentrations [5]. TAN mainly exists in two forms: unionized ammonia (NH_3_) and ionized ammonium (NH_4_^+^), with unionized ammonia being more toxic to aquatic organisms [6]. In fish, the gills are a major site of gas exchange, osmoregulation, and nitrogenous waste excretion. Because NH_3_ is highly lipid-soluble and uncharged, it can readily diffuse across the gill epithelium and enter the fish body [7], further causing tissue damage, growth inhibition, and behavioral abnormalities [8]. Existing studies have mainly focused on the adverse effects of ammonia nitrogen on fish growth, physiology, and health, as well as on strategies for controlling and regulating ammonia nitrogen concentrations in aquaculture systems. For example, Liu et al. [9] observed that rainbow trout exposed to ammonia nitrogen stress exhibited increased susceptibility to infectious hematopoietic necrosis virus (IHNV), accompanied by reduced antioxidant capacity and impaired immune function. Jin et al. [10] reported that ammonia exposure caused significant gill tissue damage, mainly characterized by gill filament fusion, epithelial cell sloughing, and chloride cell hyperplasia. Under 96 h acute ammonia nitrogen stress, red-finned pufferfish (*Takifugu rubripes*) showed significant decreases in red blood cell count, hemoglobin content, and hematocrit [11]. However, the behavioral effects of ammonia nitrogen stress in fish remain less well-characterized. Therefore, how to use abnormal fish behavior for the early warning of ammonia nitrogen stress remains an important issue in aquaculture water-quality monitoring [12].

Traditional fish behavior monitoring mainly relies on manual observation or invasive sensors. Manual observation is limited by subjectivity, poor continuity, and high labor demand, whereas invasive sensors can provide physiological data but may themselves act as stressors, making them difficult to apply in large-scale group monitoring [13]. Therefore, developing non-invasive, continuous, and quantitative methods for fish behavior monitoring is essential for the objective identification and automated analysis of fish behavioral responses [14]. In recent years, with advances in image acquisition and intelligent analysis technologies, computer vision has been increasingly applied as a non-invasive tool for studying aquatic animal behavior [15]. Computer-vision-based behavioral monitoring can automatically identify, track, and analyze continuous image sequences [16], enabling the objective extraction and quantitative characterization of fish trajectories and behavioral features, thereby providing technical support for the early identification of ammonia nitrogen stress.

Early studies on the visual monitoring of fish behavior mainly used two-dimensional image data to analyze movement trajectories, activity ranges, and local behavioral features. However, two-dimensional images are often insufficient to fully represent the actual movement state of fish in complex aquatic environments [17]. To more accurately characterize fish spatial position and movement state, researchers have gradually introduced three-dimensional vision techniques into fish behavior monitoring. For example, Cheng et al. [18] used a stereo camera combined with the DLT algorithm to achieve the high-precision reconstruction of three-dimensional fish trajectories and applied this method to abnormal water-quality identification. Xu et al. [5] analyzed the behavioral responses of three fish species under different ammonia nitrogen concentrations using an improved YOLOv8 model, and found clear differences in behavioral trajectories, activity levels, spatial distribution, and swimming speed. Mittún et al. [19] used a synchronized stereo camera system to obtain three-dimensional position, swimming speed, and nearest-neighbor distance of fish. These studies indicate that three-dimensional vision enables the quantitative characterization of fish spatial position, movement trajectories, swimming speed, and inter-individual spatial relationships. However, fish responses to ammonia nitrogen stress are multidimensional, involving not only locomotor behavior and spatial distribution, but also ventilation-related phenotypes such as ventilation activity.

Rainbow trout (Oncorhynchus mykiss) is an important cold-water aquaculture species worldwide, characterized by rapid growth, high meat quality, and substantial economic value [20]. It is also sensitive to key water-quality factors, including water temperature, dissolved oxygen, and ammonia nitrogen [21]. For rainbow trout, previous studies on ammonia nitrogen stress have mainly examined growth performance, physiological disturbance, immune response, and tissue damage, whereas non-invasive visual assessment of stress-related phenotypic changes remains less developed. At the methodological level, existing computer-vision-based studies on fish stress responses have mainly relied on body position or trajectory information to describe locomotor-related behavioral changes. However, ammonia nitrogen stress may involve not only external locomotor changes, but also ventilation-related responses that reflect a physiologically distinct dimension of stress regulation. Therefore, an integrated non-invasive visual framework that jointly characterizes locomotor and ventilatory responses would provide a more comprehensive approach for stress assessment under ammonia nitrogen exposure.

Based on this consideration, this study developed a non-invasive computer-vision-based framework for the joint characterization of rainbow trout locomotor behavior and ventilation activity under acute ammonia nitrogen stress. The main contribution of this study lies in integrating stereo-vision-based three-dimensional locomotor analysis, mouth-region optical-flow-based ventilation estimation, and multiple visual stress indicators within a unified framework. This framework enables the simultaneous analysis of locomotor and ventilatory responses under the same acute exposure process, thereby extending machine-vision-based fish stress monitoring from locomotor-focused behavioral assessment to integrated visual phenotyping of both locomotor and ventilatory responses. This study provides a reference for the objective identification of abnormal responses in rainbow trout under ammonia nitrogen stress and for the early warning of ammonia nitrogen risk in aquaculture water environments.

## 2. Materials and Methods

### 2.1. Experimental Materials

#### 2.1.1. Experimental Fish and Domestication

The rainbow trout used in this study were obtained from a breeding base in Jining, Shandong Province, China. After initial health screening, 16 rainbow trout of uniform size and apparent health were selected for the experiment, with an average body length of 14.0 ± 1.0 cm and an average body weight of 38.65 ± 2.42 g. Before the experiment, the selected experimental fish were acclimated for two weeks in a recirculating aquaculture system at the Shandong Provincial Key Laboratory of Digital Fisheries, China. During acclimation, the fish were maintained in one indoor rearing tank with dimensions of 100 × 50 × 50 cm and an approximate water volume of 250 L, corresponding to a stocking density of approximately 64 fish/m^3^, and were fed twice daily, in the morning and afternoon. The culture water was dechlorinated tap water after sufficient aeration. During rearing, one-fifth of the total water volume was replaced daily, and continuous aeration and routine tank cleaning were performed to maintain water quality. Water temperature was maintained at 16 ± 1.0 °C, dissolved oxygen was kept above 8 mg/L, pH was maintained at 7.5 ± 0.3, and TAN was kept below 0.2 mg/L. Feeding was stopped 24 h before the experiment.

#### 2.1.2. Design and Construction of the Experimental System

**Figure 1:**
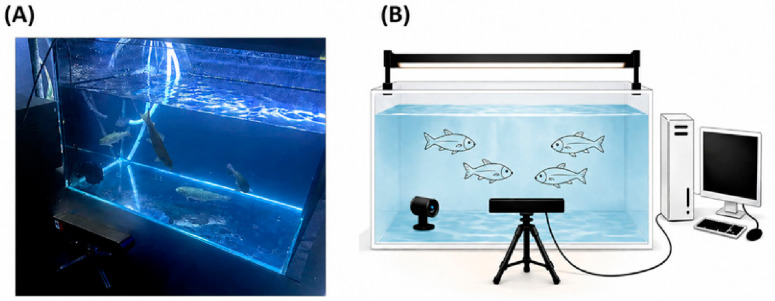
Experimental device. (**A**) Photograph of the experimental device. (**B**) Schematic diagram of the experimental device.

The experimental setup, shown in Figure 1, consisted of an aquarium, a stereo camera, a monocular camera, and a computer. The aquarium measured 60 × 40 × 40 cm, and its bottom and three side walls were covered with black background material to increase contrast and reduce glare. The stereo camera was placed facing the uncovered side of the aquarium. To minimize interference with the stereo camera’s field of view and reduce disturbance to the fish, the monocular camera was positioned in one corner inside the aquarium and directed toward the area where the rainbow trout most frequently appeared. Throughout the experiment, the camera positions and fields of view were kept unchanged. LED lights were used for supplemental illumination during video recording. To reduce the potential influence of artificial lighting on rainbow trout behavior, the fish were acclimated to LED illumination during the acclimation period.

### 2.2. Acute Stress Experiment

**Figure 2:**
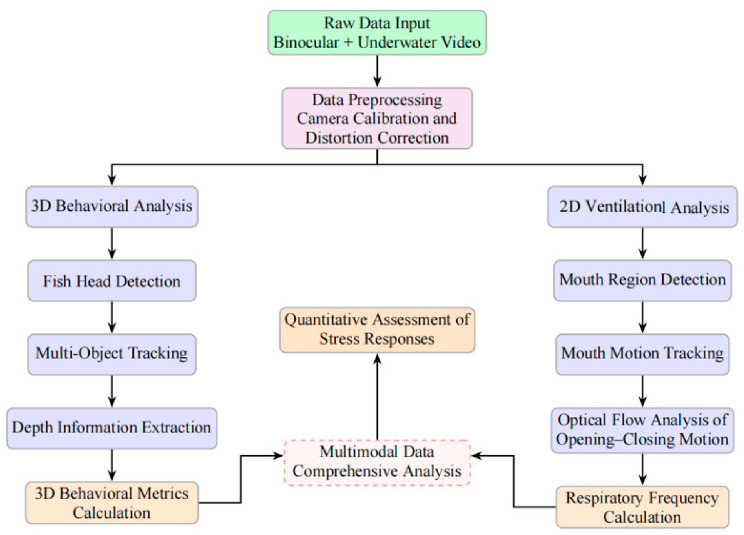
Experimental flowchart.

In this study, a stock solution was prepared using ammonium chloride (NH_4_Cl, analytical reagent grade, ≥99.5%; Tianjin Zhonglian Chemical Reagent Co., Ltd., Tianjin, China) as the ammonia nitrogen source. Based on the relevant literature and preliminary experiments, four ammonia nitrogen concentrations were established: 0, 15, 30, and 60 mg/L, with the 0 mg/L group serving as the control. Under the nominal experimental conditions (16 °C, pH 7.5), the estimated unionized ammonia nitrogen (NH_3_-N) fraction was approximately 0.92% of TAN, corresponding to NH_3_-N concentrations of 0, 0.138, 0.277, and 0.553 mg/L, respectively. This concentration gradient was designed to induce detectable acute ammonia nitrogen stress responses while avoiding mortality caused by excessive short-term exposure. The ammonia nitrogen concentration in the water was measured using a spectrophotometer (Hach Company, Loveland, CO, USA) and a reagent kit (Baishui’an Technology Co., Ltd., Suzhou, China). The acute exposure experiment was conducted in four experimental aquaria, with one aquarium assigned to each TAN treatment. For each concentration group, four rainbow trout were randomly assigned to the treatment, corresponding to a stocking density of approximately 47.6 fish/m^3^ in each aquarium. Independent samples were used across treatment groups, and no fish were reused. Each fish was considered an independent biological replicate (*n* = 4 per treatment). The sample size was selected by considering the need for independent biological replication, animal welfare, and the feasibility of synchronized stereo–monocular video acquisition under controlled acute-exposure conditions. For each TAN treatment, video data were continuously recorded for 1 h. During the initial stage of exposure, the fish typically exhibited pronounced short-term responses caused by transfer and environmental change, which could confound the assessment of ammonia-induced behavioral changes. Therefore, the final 5 min of video footage from each concentration group were selected a priori as a standardized analysis window to characterize the locomotor and ventilatory responses of rainbow trout during the relatively stable later stage of acute ammonia nitrogen exposure. The overall experimental procedure is shown in Figure 2.

### 2.3. Data Collection and Dataset Construction

The experiment used a ZED 2i stereo camera (Stereolabs, San Francisco, CA, USA) and a monocular camera (Shenzhen Baihuasheng Technology Co., Ltd., Shenzhen, China) to capture the three-dimensional locomotor behavior of rainbow trout and the opening–closing motion of the upper and lower jaw regions, respectively. The video acquisition resolutions of the stereo and monocular cameras were set to 2208 × 1242 and 1920 × 1080, respectively, and both cameras were operated at 15 fps. To synchronize the two video streams, a brief flash was used as a temporal reference at the beginning of each recording, thereby ensuring temporal alignment between the stereo and monocular videos.

**Figure 3:**
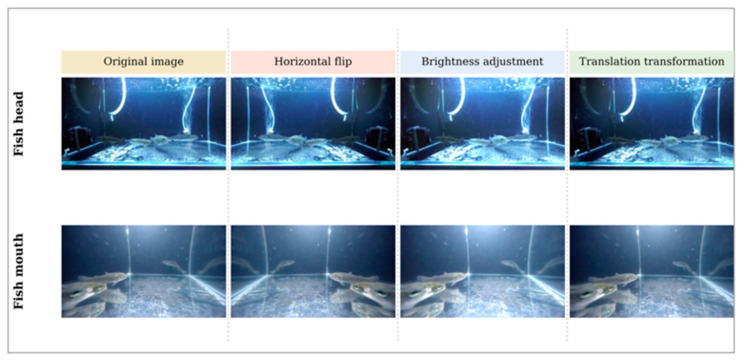
Sample images of fish head and fish mouth before and after data augmentation.

Based on the videos collected during the experiment, images were randomly extracted, and the head and mouth regions of rainbow trout were manually annotated using LabelMe software (version 5.9.1). The annotated images were used to construct datasets for three-dimensional locomotor behavior analysis and two-dimensional ventilation feature extraction. Because the background variation in the original images was limited, data augmentation was performed using horizontal flipping, brightness adjustment, and image translation. Representative original images and their augmented versions are shown in Figure 3.

After data augmentation, the three-dimensional locomotor behavior dataset contained 8200 images, whereas the two-dimensional ventilation dataset contained 4400 images. Each dataset was divided into training, validation, and test sets at a 7:2:1 ratio. Detailed information is provided in Table 1.

### 2.4. Camera Calibration and Distortion Calibration

To ensure the accuracy of three-dimensional coordinate reconstruction and ventilation frequency estimation, both cameras were calibrated and the acquired images were corrected for distortion before the experiment. For the stereo camera, Zhang’s calibration method [22] was used within the OpenCV framework to obtain the intrinsic matrices of the left and right cameras and to determine the extrinsic parameters between the two views. The camera intrinsic matrix K is given in Equation (1).

(1) $$K=\left[\begin{array}{ccc} f_{x} & 0 & c_{x}\\ 0 & f_{y} & c_{y}\\ 0 & 0 & 1 \end{array}\right]$$

Here, $f_{x}$ and $f_{y}$ represent the horizontal and vertical focal lengths, respectively, and ($c_{x}$, $c_{y}$) are the coordinates of the principal point. The extrinsic relationship between the left and right cameras is shown in Equation (2).

(2) $$X_{r}=RX_{l}+T$$

Here, $X_{l}$ and $X_{r}$ represent the three-dimensional coordinates of the same spatial point in the left and right camera coordinate systems, respectively. *R* is the rotation matrix that transforms coordinates from the left camera coordinate system to the right camera coordinate system; and *T* is the corresponding translation vector.

To reduce the effect of lens distortion on object detection and spatial localization accuracy, the images were corrected using radial and tangential distortion models, as defined in Equation (3) and Equation (4), respectively.

(3) $$\left\{\begin{array}{l} x_{\mathrm{corrected}}=x\left(1+k_{1}r^{2}+k_{2}r^{4}+k_{3}r^{6}\right),\\ y_{\mathrm{corrected}}=y\left(1+k_{1}r^{2}+k_{2}r^{4}+k_{3}r^{6}\right) \end{array}\right.$$

(4) $$\left\{\begin{array}{l} x_{\mathrm{corrected}}=x+\left[2p_{1}\mathrm{xy}+p_{2}\left(r^{2}+2x^{2}\right)\right],\\ y_{\mathrm{corrected}}=y+\left[p_{1}\left(r^{2}+2y^{2}\right)+2p_{2}\mathrm{xy}\right] \end{array}\right.$$

Here, (x,y) represent the normalized image-plane coordinates of the target point before distortion correction, and ($x_{\mathrm{corrected}}$, $y_{\mathrm{corrected}}$) represent the corresponding normalized coordinates after correction. *r* represents the radial distance from the target point to the principal point on the normalized image plane, $r^{2}\;$= *x*^2^ + *y*^2^; *k*_1_, *k*_2_, and *k*_3_ are the radial distortion coefficients, whereas $p_{1}$ and $p_{2}$ are the tangential distortion coefficients.

The corrected images were used for fish-head detection and three-dimensional coordinate reconstruction to improve the spatial consistency between the two-dimensional detection results and the depth information. For the monocular camera, intrinsic calibration and distortion correction were also performed to improve the stability of mouth-region localization and optical-flow feature extraction.

### 2.5. Object Detection Framework Based on YOLOv11

**Figure 4:**
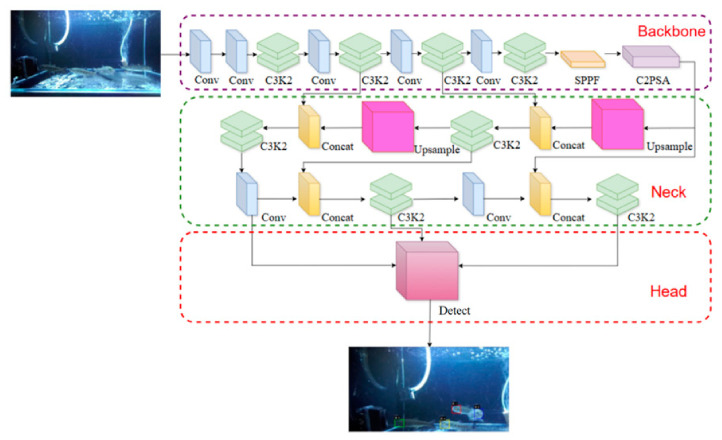
Network architecture of YOLOv11.

YOLO (You Only Look Once) is a representative single-stage object detection method with end-to-end training, fast inference, and strong real-time performance, and has been widely used in object detection tasks. Among the YOLO series, YOLOv11 provides a favorable balance between detection accuracy and computational efficiency. Therefore, YOLOv11 was used to construct the object detection model in this study. The overall network architecture of YOLOv11 is shown in Figure 4, consisting of a backbone network, a feature-fusion module, and a detection head. Model training was performed on a workstation equipped with an NVIDIA GPU. The maximum number of training epochs was set to 100, and an early stopping strategy was applied to reduce the risk of overfitting. Training was automatically stopped when the validation performance did not improve for 15 consecutive epochs. The batch size was set to 8, and the input image resolution was 640 × 640 pixels. This configuration improved the training efficiency and computational stability while retaining sufficient object details for detection.

In this study, YOLOv11n was selected as the object detection model because of its lightweight architecture and computational efficiency, which are suitable for continuous video processing. This configuration provided a practical balance between real-time performance and detection accuracy while meeting the computational requirements of three-dimensional coordinate extraction and optical-flow-based ventilation analysis.

### 2.6. Processing of Locomotor Behavior Data

**Figure 5:**
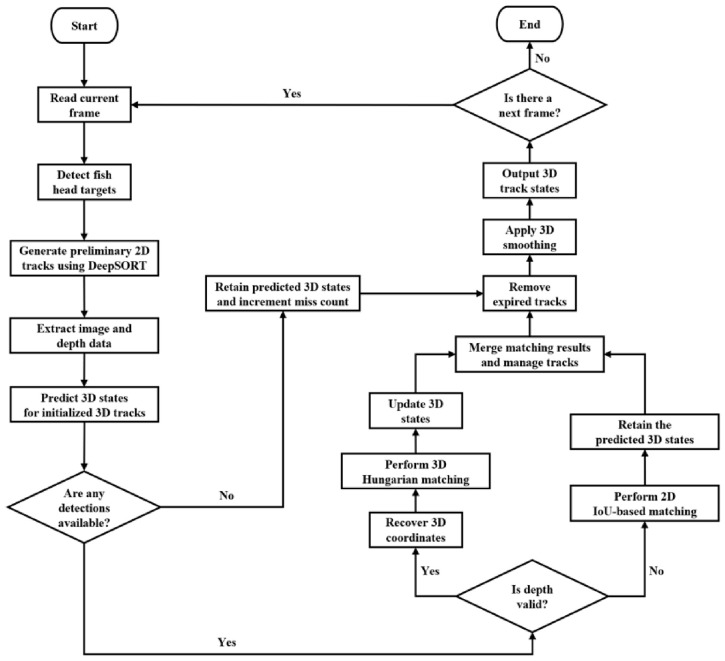
Overall flowchart of the hybrid 2D–3D tracking process.

Using videos of rainbow trout captured by the stereo camera, the three-dimensional spatiotemporal positions of the fish were obtained by integrating object detection, multi-object tracking, and depth mapping. Locomotor indicators, including amount of exercise, average swimming speed, and spatial distribution, were then calculated to evaluate the effects of ammonia nitrogen stress on rainbow trout locomotor behavior. Therefore, this study used DeepSORT as a two-dimensional trajectory maintenance framework and combined it with an observation-quality-guided 2D–3D hybrid association strategy to develop a multi-object tracking method for rainbow trout. In this method, DeepSORT was used to maintain the continuity of two-dimensional trajectories in the image plane. On this basis, three-dimensional state prediction, observation update, and trajectory correction were performed to improve the stability of trajectory association and three-dimensional position reconstruction in complex scenes. The overall workflow of this method is shown in Figure 5.

#### 2.6.1. Observation-Trajectory Matching Based on a Hybrid Association Strategy

**Figure 6:**
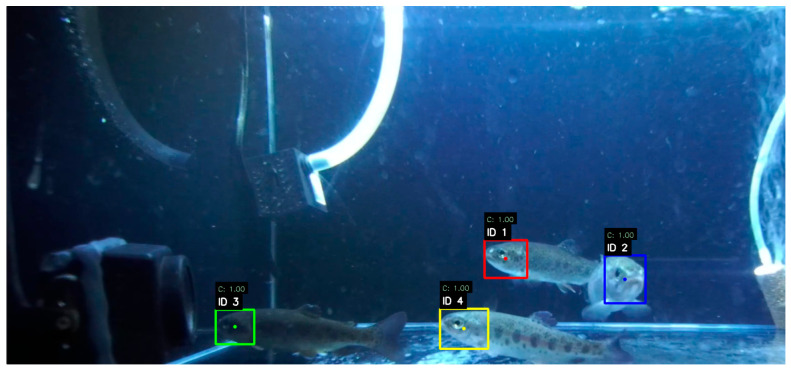
Visualization of multi-object tracking results for rainbow trout.

In multi-object scenarios, target observations are susceptible to occlusion, detection errors, and missed detections, and three-dimensional coordinates may not be stably obtained when depth information is invalid or missing. To maintain target identity continuity, a 2D–3D hybrid association strategy based on observation-quality switching was applied. For observations with valid depth information, three-dimensional spatial information was prioritized for observation-to-trajectory association. For observations with invalid depth information, the system fell back to two-dimensional auxiliary association to maintain trajectory continuity. After matching, the two sets of association results were integrated, with the 3D matching results based on valid depth observations assigned higher priority. Unmatched trajectories were retained or deleted according to predefined rules, whereas unmatched observations were initialized as new candidate trajectories. A visualization of multi-object tracking for rainbow trout is shown in Figure 6.

For targets with valid depth information, a three-dimensional association cost matrix was constructed using the spatial distance between the reconstructed three-dimensional observation coordinates and the predicted state of the corresponding historical trajectory. The Hungarian algorithm was then used to perform observation-to-trajectory association. The association cost in this mode was calculated using Equation (5).

(5) $$C_{\mathrm{ij}}^{3D}={\left\|z_{i,k}^{3D}-{\hat{p}}_{j,k|k-1}\right\|}_{2}$$

Here, $C_{\mathrm{ij}}^{3D}$ represents the three-dimensional association cost between the i-th valid 3D observation in the k-th frame and the j-th historical trajectory. $z_{i,k}^{3D}$ represents the 3D observation coordinates of the i-th target in the current frame, and ${\hat{p}}_{j,k|k-1}$ represents the predicted three-dimensional position of the j-th trajectory in the current frame.

For targets with invalid depth information, the system enters the two-dimensional auxiliary association process. Only trajectories that are successfully associated in 2D mode retain their current three-dimensional predicted states, without performing an observation update. An auxiliary association cost matrix was constructed based on the intersection over union (IoU) between the current detection box and the two-dimensional projected box of the historical trajectory. This matrix was calculated using Equation (6).

(6) $$\begin{array}{c} C_{\mathrm{ij}}^{2D}=1-\mathrm{IoU}\left(B_{i}^{\det},\;B_{j}^{\mathrm{trk}}\right) \end{array}$$

Here, $C_{\mathrm{ij}}^{2D}$ represents the two-dimensional auxiliary association cost between the i-th two-dimensional detection bounding box in the k-th frame and the j-th historical trajectory; IoU represents the intersection-over-union ratio of the two bounding boxes; $B_{i}^{\det}$ represents the two-dimensional detection bounding box of the i-th object in the current frame; and $B_{j}^{\mathrm{trk}}$ represents the predicted bounding box of the j-th trajectory in the image plane.

#### 2.6.2. Three-Dimensional State Estimation

Three-dimensional observations are susceptible to detection jitter, depth-estimation noise, and transient positioning anomalies, which may lead to local fluctuations in the trajectory over time. To improve the continuity and stability of the three-dimensional position sequence, an improved three-dimensional Kalman filter was used for recursive target-state estimation. For trajectories that had completed three-dimensional initialization, the state was predicted from the previous time step and updated when reliable three-dimensional observations were available. It should be noted that three-dimensional state prediction and update were performed only for trajectories with completed three-dimensional initialization. For new observations without valid depth information, three-dimensional initialization was not performed immediately; instead, they were first treated as two-dimensional candidate trajectories to maintain trajectory continuity. Three-dimensional initialization was completed, and the corresponding trajectories were incorporated into the state estimation process, only after reliable depth observations were subsequently obtained.

Based on the above recursive process, a state-space model incorporating three-dimensional position, velocity, and acceleration was constructed to describe the short-term motion state of the target. The calculation of the target state vector $x_{k}$ at frame k is shown in Equation (7).

(7) $$x_{k}={\left[X_{k},{\;Y}_{k},{\;Z}_{k},{\;V}_{x,k},{\;V}_{y,k},\;V_{z,k},{\;A}_{x,k},{\;A}_{y,k},\;A_{z,k}\right]}^{T}$$

Here, $\left(X_{k},{\;Y}_{k},{\;Z}_{k}\right)$ represent the three-dimensional spatial coordinates of the target in the k-th frame, ($V_{x,k},\;V_{y,k},\;V_{z,k}$) represent the velocity components in the corresponding directions, and ($A_{x,k},{\;A}_{y,k},{\;A}_{z,k}$) represent the corresponding acceleration components. The state transition equation of the system is shown in Equation (8).

(8) $$x_{k}=Fx_{k-1}+w_{k}$$

Here, F represents the state transition matrix, and $w_{k}$ represents the process noise. When a reliable 3D observation is available in the current frame, the observation vector $z_{k}$ for the k-th frame is calculated as shown in Equation (9).

(9) $$z_{k}={\left[X_{k}^{\mathrm{obs}},{\;Y}_{k}^{\mathrm{obs}},{\;Z}_{k}^{\mathrm{obs}}\right]}^{T}$$

Here, ($X_{k}^{\mathrm{obs}},\;Y_{k}^{\mathrm{obs}},\;Z_{k}^{\mathrm{obs}}$) represent the three-dimensional observation coordinates reconstructed from the current frame. The corresponding observation model equations are given in Equation (10).

(10) $$z_{k}=Hx_{k}+v_{k}$$

Here, H represents the observation matrix, and $v_{k}$ represents the observation noise.

To reduce the influence of abnormal depth values and sudden positioning errors on state updates, an outlier detection procedure was performed before the update step based on the Mahalanobis distance between the predicted state and the current observation. When the Mahalanobis distance exceeded a predefined threshold, the current observation was identified as an outlier and excluded from the state update; otherwise, the observation was used to correct the predicted state. The Mahalanobis distance $d_{k}$ can be expressed as in Equation (11).

(11) $$d_{k}=\sqrt{{\left(z_{k}-H{\hat{x}}_{k|k-1}\right)}^{T}S_{k}^{-1}\left(z_{k}-H{\hat{x}}_{k|k-1}\right)}$$

Here, ${\hat{x}}_{k|k-1}$ represents the predicted state vector for frame k, and $S_{k}^{-1}$ represents the inverse of the innovation covariance matrix $S_{k}$. The calculation of the innovation covariance matrix $S_{k}$ is given in Equation (12).

(12) $$S_{k}=HP_{k|k-1}H^{T}+R_{k}$$

Here, $P_{k|k-1}$ represents the prediction state covariance matrix, and $R_{k}$ represents the observation noise covariance matrix.

Because observation quality may vary over time, the observation noise covariance matrix $R_{k}$ was adaptively adjusted according to the dispersion of recent measurements, thereby improving the filter’s ability to handle variations in observation reliability. After state estimation, short-window mean smoothing was applied to the three-dimensional position sequence because local short-term fluctuations may still remain in the recursively estimated trajectory. This smoothing step reduced local jitter while preserving the overall movement trend, thereby improving the continuity and stability of the trajectory sequence and the reliability of subsequent locomotor indicator calculations.

#### 2.6.3. Reconstruction of the Three-Dimensional Position of the Fish Head

**Figure 7:**
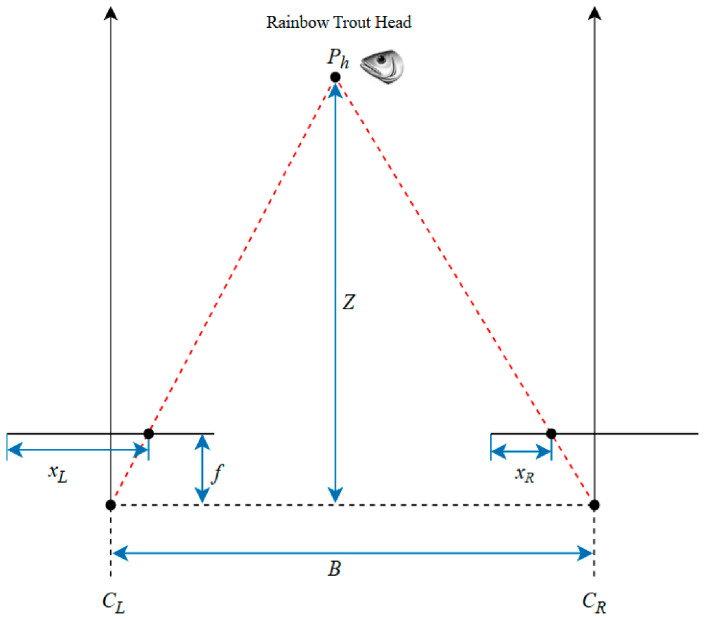
Schematic diagram of the fish head 3D reconstruction principle.

After stable trajectory association and three-dimensional state estimation were obtained, the three-dimensional position of the trout head was reconstructed based on stereo vision. Figure 7 shows the geometric relationship of stereo imaging, where *C_L_* and *C_R_* are the optical centers of the left and right cameras, *B* is the baseline length, *f* is the focal length, and *P_h_* is the spatial point of the fish head. The x-coordinates of the projected points of this spatial point on the left and right imaging planes are $x_{L}$ and $x_{R},\;$ respectively, and the difference between the two is defined as the disparity d.

After stereo calibration and rectification were completed, the target depth was recovered according to the stereo geometric relationship. The pixel coordinates (u, v) of the fish head feature points on the left image plane in Figure 7 were then mapped to the three-dimensional spatial coordinates (*X*, *Y*, *Z*) using the camera intrinsic parameters. The mapping relationship is shown in Equations (13)–(15).

(13) $$X=\frac{(u-c_{x})Z}{f_{x}}$$

(14) $$Y=\frac{(v-c_{y})Z}{f_{y}}$$

(15) $$Z=\frac{f_{x}B}{d}$$

Here, *f_x_* and *f_y_* represent the camera’s equivalent focal lengths in the horizontal and vertical directions, respectively, and ($c_{x}$,${\;c}_{y}$) are the coordinates of the principal point.

Based on the mapping relationships described above, the sequence of fish three-dimensional positions was obtained across consecutive video frames. During the experiment, the stereo camera position remained fixed, and all three-dimensional coordinates were expressed in the same coordinate system, thereby ensuring the comparability of rainbow trout three-dimensional behavioral data across different experimental conditions.

#### 2.6.4. Evaluation Indicators

Based on the three-dimensional position sequences of rainbow trout in consecutive video frames, locomotor indicators, including amount of exercise, average swimming speed, and spatial distribution, were calculated to characterize the behavioral responses of rainbow trout under different ammonia nitrogen concentrations. Let the three-dimensional coordinates of the i-th fish in the t-th frame be $\left(x_{t},\;y_{t},{\;z}_{t}\right)$, where the video frame rate is f and the time interval between adjacent frames is Δt; this is calculated as shown in Equation (16).

(16) $$\Delta t=\frac{1}{f}$$

(1) Amount of exercise

The amount of exercise was used to quantify the cumulative movement of each fish over a given period, reflecting its spatial activity range and movement persistence. This indicator can help distinguish low-activity and high-activity states and is suitable for assessing activity inhibition or stress-induced hyperactivity under stress conditions. The amount of exercise was calculated as the sum of frame-to-frame three-dimensional displacements, as shown in Equation (17).

(17) $$D=\sum_{t=2}^{T}\sqrt{(x_{t}-x_{t-1}{)}^{2}+(y_{t}-y_{t-1}{)}^{2}+(z_{t}-z_{t-1}{)}^{2}}$$

Here, D represents the total distance traveled by the fish over the entire observation period, and T represents the effective tracking time.

(2) Average swimming speed

Average swimming speed was used to quantify the overall locomotor level of the fish during the observation period, thereby reflecting its swimming capacity under different ammonia nitrogen concentrations. The instantaneous velocity between two adjacent frames is denoted as $v_{t}$, and is calculated as shown in Equation (18).

(18) $$v_{t}=\frac{\sqrt{(x_{t}-x_{t-1}{)}^{2}+(y_{t}-y_{t-1}{)}^{2}+(z_{t}-z_{t-1}{)}^{2}}}{\Delta t}$$

On this basis, the average swimming speed $\bar{v}$ over the entire valid tracking period is calculated using Equation (19).

(19) $$\bar{v}=\frac{1}{T}\sum_{t=1}^{T}v_{t}$$

(3) Spatial distribution

Spatial distribution was used to characterize the spatial occupancy patterns and activity preferences of fish within the experimental area. Based on the projected coordinates of rainbow trout three-dimensional trajectories on a specified plane, the observation area was divided into regular grids. The number of trajectory points within each grid was counted, and its proportion relative to the total number of trajectory points was calculated to construct a normalized spatial distribution matrix. This proportion was calculated using Equation (20).

(20) $$P_{\mathrm{ij}}=\frac{N_{\mathrm{ij}}}{\sum_{p=1}^{a}\sum_{q=1}^{b}N_{\mathrm{pq}}}$$

Here, $P_{\mathrm{ij}}$ represents the proportion of trajectory points within grid (*i*, *j*) relative to the total number of trajectory points; the sum of $P_{\mathrm{ij}}$ across all grids is 1. $N_{\mathrm{ij}}$ represents the number of trajectory points in the corresponding grid, whereas a and b represent the number of subdivisions of the grid in the two coordinate directions, respectively.

To further quantify the group-level spatial relationship of rainbow trout, the three-dimensional mean inter-individual distance was calculated as a supplementary spatial indicator. For each frame, the Euclidean distance between each available pair of fish was calculated based on the reconstructed three-dimensional coordinates. This distance was calculated using Equation (21).

(21) $$L_{ij,t}=\sqrt{{\left(X_{i,t}-X_{j,t}\right)}^{2}+{\left(Y_{i,t}-Y_{j,t}\right)}^{2}+{\left(Z_{i,t}-Z_{j,t}\right)}^{2}}$$

where $L_{ij,t}$ represents the three-dimensional distance between fish i and fish j in the t-th frame, and ($X_{i,t}$, $Y_{i,t}$, $Z_{i,t}$) and ($X_{j,t}$, $Y_{j,t}$, $Z_{j,t}$) represent the reconstructed three-dimensional coordinates of fish i and fish j, respectively.

### 2.7. Processing of Ventilation Frequency Data

In addition to locomotor behavior, ventilation frequency is also a key indicator of fish responses to ammonia nitrogen stress. Ventilation frequency is closely related to metabolic oxygen demand. Under environmental stress, fish often regulate their ventilation rhythm to meet increased oxygen requirements and maintain homeostasis [23]. Computer-vision-based non-contact analysis provides a technical basis for continuously quantifying fish ventilation activity. Based on this, object detection and optical-flow analysis were combined to analyze the mouth opening–closing movements of rainbow trout in underwater videos and to estimate ventilation frequency.

#### 2.7.1. Principles and Applications of the Optical Flow Method

The optical flow method is used to estimate the apparent motion of objects between adjacent frames in a video sequence. Based on fundamental assumptions such as brightness constancy, temporal continuity, and spatial consistency, this method enables the extraction of local motion features from images. Depending on the constraint strategy used, classical optical flow methods can be broadly categorized into local and global approaches. Among the local optical flow methods, the Farnebäck method [24] estimates pixel displacement between adjacent frames by performing polynomial expansion in local image neighborhoods, thereby capturing small-amplitude and continuous local motion changes. Because the mouth opening–closing movement of rainbow trout is characterized by small amplitude, strong localization, and clear periodicity, the Farnebäck local optical flow method was selected to analyze the motion changes in the fish’s mouth region.

The key to optical-flow analysis is to quantify the temporal changes in the mouth opening–closing motion. YOLOv11n was used to localize the fish mouth region in consecutive video frames, and the detected mouth region was divided into upper- and lower-jaw regions. Because the ventilation-related mouth motion of rainbow trout occurs mainly in the vertical direction, the average vertical optical-flow component was calculated separately for each region. This strategy emphasizes the relative motion between the upper and lower jaws while reducing the influence of whole-body sway, local drift, and background noise. During the mouth-opening phase, the vertical optical-flow components of the upper- and lower-jaw regions generally indicate opposite vertical motion; during the mouth-closing phase, they indicate convergent vertical motion. Based on these temporal characteristics, a phase-discrimination method with time-threshold constraints was used to identify ventilation events and estimate the ventilation frequency of rainbow trout.

#### 2.7.2. Calculation of Ventilation Frequency

Ventilation frequency refers to the number of times the fish’s mouth opens and closes per unit of time (usually per minute), and is calculated as shown in Equation (22).

(22) $$\begin{array}{c} \mathrm{Ventilation}\;\mathrm{frequency}=\frac{N_{\mathrm{vent}}}{T_{\mathrm{valid}}} \end{array}$$

Here, $N_{\mathrm{vent}}$ represents the number of times the fish’s mouth was detected opening and closing, whereas $T_{\mathrm{valid}}$ represents the total effective duration during which the fish’s mouth region was successfully detected and used for ventilation analysis.

### 2.8. Model Evaluation Metrics

To evaluate the performance of the fish-head and fish-mouth detection models, commonly used object detection metrics, including precision, recall, average precision (AP), and mean average precision (mAP), were adopted.

Precision represents the proportion of correctly detected targets among all predicted targets, whereas recall represents the proportion of correctly detected targets among all ground-truth targets. These metrics were calculated using Equations (23) and (24).

(23) $$\begin{array}{c} \mathrm{Precision}=\frac{\mathrm{TP}}{\mathrm{TP}+\mathrm{FP}} \end{array}$$

(24) $$\begin{array}{c} \mathrm{Recall}=\frac{\mathrm{TP}}{\mathrm{TP}+\mathrm{FN}} \end{array}$$

Here, TP represents the number of true positives, that is, targets that were correctly detected; FP represents the number of false positives, that is, targets that were incorrectly detected; and FN represents the number of false negatives, that is, targets that were present but not detected.

Average precision (AP) was used to evaluate the overall detection performance of the model for a single class. It corresponds to the area under the precision–recall curve and was calculated using Equation (25).

(25) $$\mathrm{AP}=\int_{0}^{1}P(R)dR$$

Here, P(R) represents the precision value corresponding to recall R. mAP represents the mean of AP values across all classes, as shown in Equation (26).

(26) $$\mathrm{mAP}=\frac{1}{N}\sum_{i=1}^{N}AP_{i}$$

Here, N represents the number of categories, and AP*_i_* represents the average precision for the i-th category.

### 2.9. Statistical Analysis

Statistical analyses were performed using Python software (version 3.11) with the SciPy and statsmodels libraries. The effects of ammonia nitrogen concentration on locomotor and ventilation indicators were analyzed using one-way analysis of variance (ANOVA), followed by Tukey’s HSD test for multiple comparisons when significant differences were detected. Each fish was treated as one biological replicate. Statistical significance was set at *p* < 0.05. Quantitative results are presented as individual values, summary statistics, or mean ± standard deviation, as appropriate.

## 3. Results

### 3.1. Model Performance

To verify whether the front-end detection module could provide reliable inputs for subsequent fish locomotor behavior and ventilation analysis, the fish-head and fish-mouth detection models were evaluated separately.

**Figure 8:**
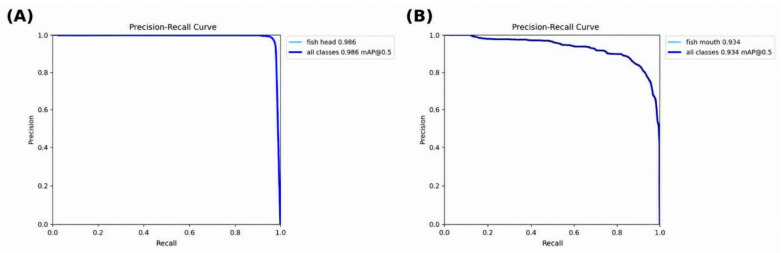
Precision–recall curves of the detection models. (**A**) Fish head detection model. (**B**) Fish mouth detection model.

The detection performance of the fish-head and mouth-region models is shown in Figure 8 and Table 2. The fish-head detection model achieved precision, recall, mAP@0.5, and mAP@0.5:0.95 values of 0.986, 0.963, 0.986, and 0.695, respectively. The corresponding values for the mouth-region detection model were 0.836, 0.888, 0.934, and 0.653, respectively. Overall, the mouth-region detection model performed slightly worse than the fish-head detection model, which may be related to the smaller size of the mouth region, less distinct edge features, and substantial appearance changes during mouth opening and closing. Nevertheless, the detection performance of both models was sufficient to support subsequent fish-head tracking and mouth-region optical-flow analysis.

### 3.2. Locomotor Responses of Rainbow Trout Under Acute Ammonia Nitrogen Stress

#### 3.2.1. Amount of Exercise

Table 3 presents the amount of exercise of rainbow trout under different ammonia nitrogen concentrations. The group-level amount of exercise was highest in the control group, reaching 2582.86 cm, whereas the values in the 15, 30, and 60 mg/L groups were 1411.56, 1781.11, and 1078.59 cm, respectively. Overall, the ammonia nitrogen treatment groups showed lower total exercise amounts than the control group, with the lowest value observed in the 60 mg/L group. Although the amount of exercise showed an overall decreasing tendency under ammonia nitrogen exposure, the differences among groups were not statistically significant (*p* > 0.05).

#### 3.2.2. Average Swimming Speed and Its Temporal Variation Characteristics

**Figure 9:**
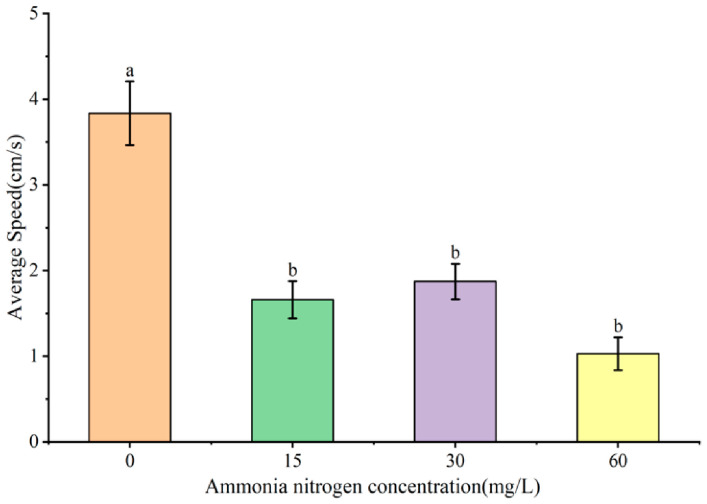
Average swimming speed of rainbow trout under different ammonia nitrogen concentrations. Different letters above the bars (a, b) indicate significant differences among groups (Tukey’s HSD, *p* < 0.05).

Figure 9 shows the average swimming speeds of rainbow trout under different ammonia nitrogen concentrations. The average swimming speed was highest in the control group (3.83 cm/s), decreased to 1.66 cm/s in the 15 mg/L group, slightly increased to 1.87 cm/s in the 30 mg/L group, and reached the lowest value in the 60 mg/L group (1.03 cm/s). One-way analysis of variance (ANOVA) showed that ammonia nitrogen concentration had a significant effect on average swimming speed (*F* = 9.34, *p* = 0.0023). Tukey’s HSD post hoc test indicated that the control group was significantly higher than the three ammonia nitrogen treatment groups (*p* < 0.05), whereas no significant differences were observed among the 15, 30, and 60 mg/L groups (*p* > 0.05).

**Figure 10:**
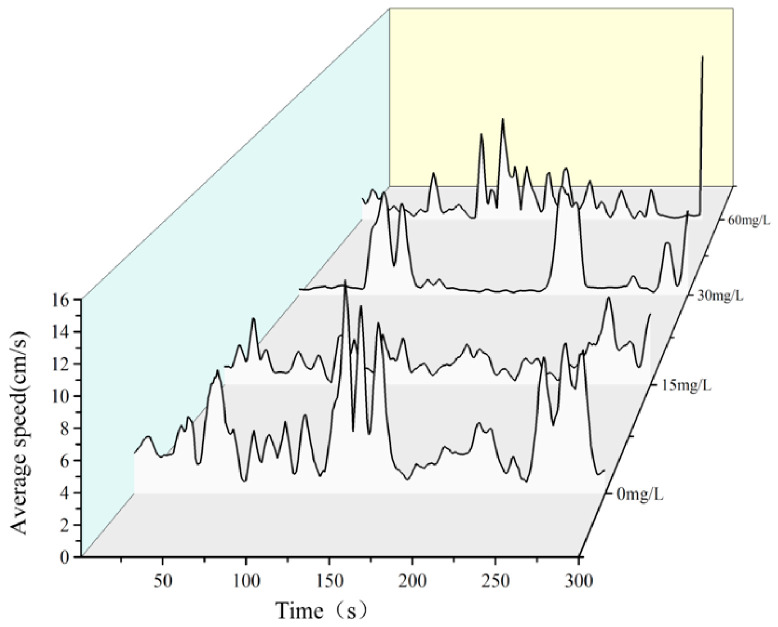
Changes in the average swimming speed of rainbow trout over time under different ammonia nitrogen concentrations.

Figure 10 shows the temporal changes in average swimming speed of the rainbow trout under different ammonia nitrogen concentrations during the observation period. The control group showed pronounced speed fluctuations throughout the observation period. The 15 mg/L group also showed clear fluctuations, and swimming speed remained relatively high during some periods. In contrast, the 30 mg/L group showed a smoother curve with fewer local peaks. The 60 mg/L group was characterized mainly by low-speed fluctuations, with only occasional short-term increases. Overall, different ammonia nitrogen concentrations not only reduced rainbow trout locomotor activity, but also altered the temporal variation and dynamic response patterns of average swimming speed.

#### 3.2.3. Spatial Distribution

**Figure 11:**
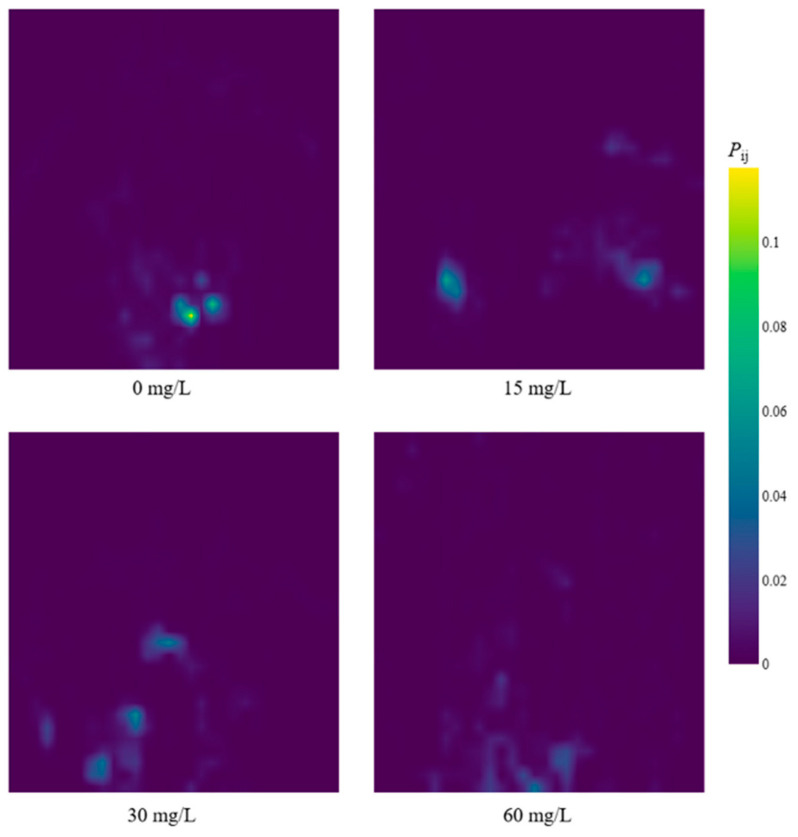
Heatmaps of the spatial occupancy distribution of rainbow trout in the X–Y plane under different ammonia nitrogen concentrations.

The spatial distribution heatmap on the X–Y plane (Figure 11) further illustrates differences in the spatial occupancy of rainbow trout in the experimental aquarium under different ammonia nitrogen concentrations. In the control group, high-density activity regions were relatively concentrated, mainly in the lower region of the X–Y plane. In the 15 mg/L group, several high-density regions were still observed, but the hotspot distribution became more dispersed than that in the control group. In the 30 mg/L group, the high-density regions showed a more evident multi-point distribution pattern. In the 60 mg/L group, the high-density activity regions were less concentrated and showed a more scattered spatial occupancy pattern.

To further quantify group-level spatial relationships, the three-dimensional mean inter-individual distance was calculated as a supplementary spatial indicator. The mean inter-individual distance decreased from 15.36 cm in the control group to 14.22, 12.54, and 11.45 cm in the 15, 30, and 60 mg/L groups, respectively. This decreasing pattern suggests a tendency toward reduced group spacing under ammonia nitrogen exposure. However, no significant difference was observed among groups (*p* > 0.05). These results indicate that ammonia nitrogen exposure altered the spatial occupancy pattern of rainbow trout, while the three-dimensional inter-individual distance provided supplementary descriptive information on group spacing rather than a primary concentration-sensitive indicator.

Taken together, rainbow trout showed changes in amount of exercise, average swimming speed, and spatial distribution under ammonia nitrogen exposure. Among these locomotor indicators, average swimming speed showed the clearest response to ammonia nitrogen concentration.

### 3.3. Ventilatory Responses of Rainbow Trout Under Acute Ammonia Nitrogen Stress

#### 3.3.1. Visual Representation of Mouth-Region Optical-Flow Signals for Ventilation Analysis

**Figure 12:**
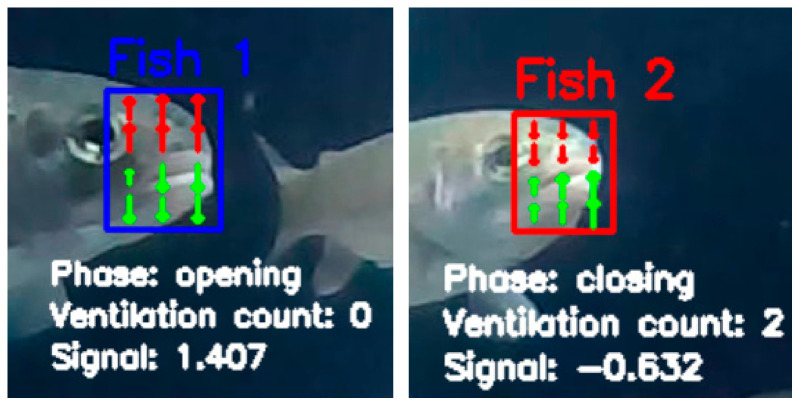
Representative optical flow patterns in the mouth region during the opening and closing phases of ventilation. Blue and red boxes indicate detected mouth regions of different fish, and red and green arrows indicate optical-flow vectors in the upper- and lower-jaw regions, respectively.

**Figure 13:**
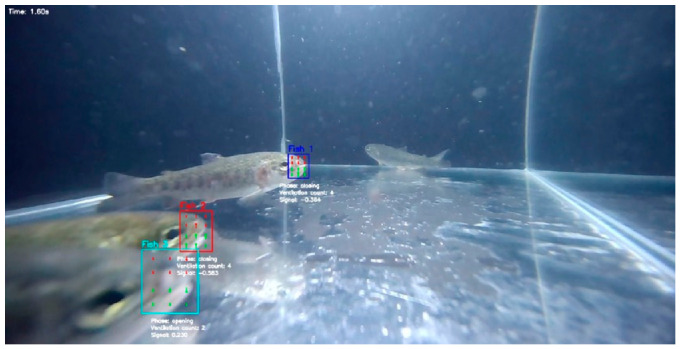
Visualization of mouth detection and analysis in rainbow trout.

Figure 12 presents the distribution characteristics of vertical optical flow in the rainbow trout mouth region during the mouth opening and closing phases of ventilation. The vertical optical-flow vectors reflected the local movement of the upper- and lower-jaw regions during ventilation. Figure 13 shows representative visualization results of the mouth-region analysis.

**Figure 14:**
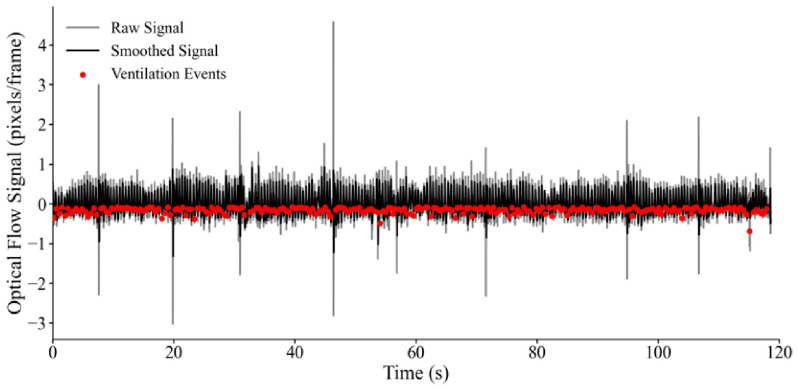
Time-series visualization of the ventilation signal of rainbow trout derived from optical flow analysis.

Time-series data of ventilation signals from a representative fish over an approximately 120 s recording period were selected for analysis. The ventilation signal and the detected ventilation events are shown in Figure 14. The raw signal showed periodic fluctuations, while the smoothed signal retained the main rhythmic pattern of mouth opening and closing.

**Figure 15:**
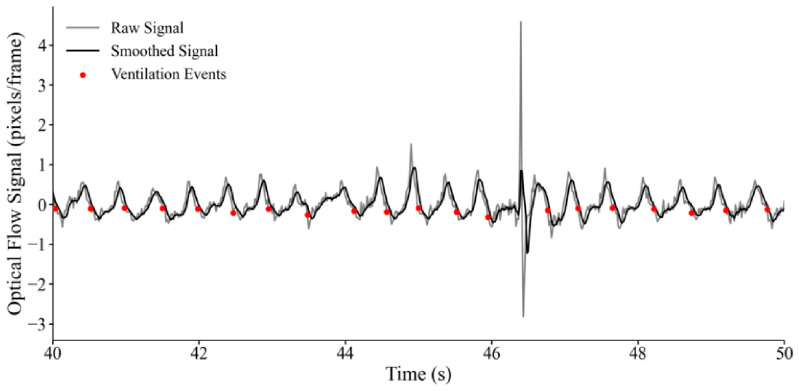
Enlarged view of the ventilation signal of rainbow trout derived from optical flow analysis during 40–50 s.

A local magnification of the 40–50 s interval in Figure 14 is shown in Figure 15. The ventilation event markers were mainly located after each positive fluctuation, near the transition from positive to negative signal values, indicating that the recognition strategy could accurately capture the key transition points of mouth opening–closing motion during rainbow trout ventilation.

#### 3.3.2. Validation of Ventilation Frequency Extraction Accuracy

To verify the accuracy of the automated ventilation-frequency extraction results, 50 video clips were randomly selected from the ventilation-analysis videos, covering different TAN concentration groups and different individuals. The selected clips ranged in duration from 30 s to several minutes and contained clearly visible mouth opening–closing movements. A complete mouth opening–closing cycle was defined as one ventilation cycle, and two observers independently counted the ventilation events without referring to the algorithm outputs. For segments with inconsistent counts, the final manual reference value was determined after rechecking and confirmation. The algorithm-derived counts were then compared with the manual counts. The results showed good agreement between the automated and manual measurements, with an R^2^ of 0.9848 based on linear regression and a mean counting accuracy of approximately 92%, indicating that the proposed method could effectively capture changes in the ventilation frequency of rainbow trout.

#### 3.3.3. Changes in the Ventilation Frequency of Rainbow Trout at Different Ammonia Nitrogen Concentrations

**Figure 16:**
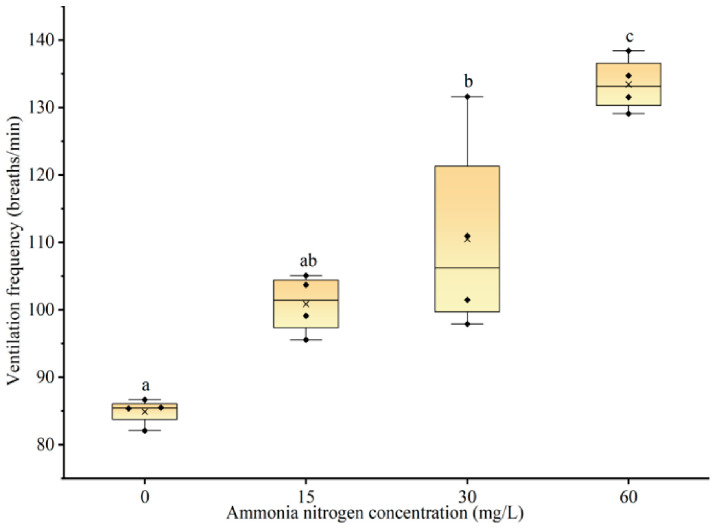
Boxplots of ventilation frequency in rainbow trout under different ammonia nitrogen concentrations. Different letters above the boxes indicate significant differences among groups according to Tukey’s HSD test (*p* < 0.05). Black diamonds indicate individual observations, and × symbols indicate mean values.

As the ammonia nitrogen concentration increased, the ventilation frequency of rainbow trout showed a gradual upward trend (Figure 16). The average ventilation frequency increased from 84.91 breaths/min in the control group to 100.87, 110.48, and 133.43 breaths/min in the 15, 30, and 60 mg/L groups, respectively. One-way analysis of variance (ANOVA) showed that ammonia nitrogen concentration had a highly significant effect on the ventilation frequency of rainbow trout (*F* = 24.63, *p* < 0.001). Tukey’s HSD post hoc test further indicated that ventilation frequency in the 30 and 60 mg/L groups was significantly higher than that in the control group (*p* < 0.05). The 60 mg/L group was also significantly higher than the other groups, whereas the 15 mg/L group did not differ significantly from either the control group or the 30 mg/L group.

## 4. Discussion

Ammonia nitrogen is one of the most common stressors in aquaculture water environments. Its effects on fish are reflected not only in physiological disturbances, such as tissue damage, metabolic disorders, and altered ventilation rhythms, but also in behavioral alterations, including reduced swimming activity and abnormal spatial distribution [4]. In this study, rainbow trout showed simultaneous enhancement of ventilation activity and suppression of locomotor behavior under acute ammonia nitrogen stress, indicating that ammonia nitrogen stress did not simply reduce overall activity level, but instead induced differentiated responses in ventilation regulation and locomotor control. From the perspective of fish physiological regulation, ammonia can disrupt acid–base balance and ionic homeostasis, thereby interfering with normal physiological functions [4]. It can also induce compensatory ventilation regulation in response to external ammonia exposure [25]. In parallel, ammonia nitrogen stress may be associated with neurological dysfunction, which can further affect locomotor control and behavioral state [26]. Therefore, the responses of rainbow trout to acute ammonia nitrogen stress are better understood from two complementary perspectives: ventilation compensation and disrupted locomotor regulation.

We observed a sustained increase in ventilation frequency in rainbow trout under acute ammonia nitrogen stress. This increase may reflect the early activation of physiological compensation. Once ammonia enters the fish body, it can cause fluctuations in plasma pH and disturb the homeostasis of ions such as Na^+^ and Cl^−^, thereby increasing the homeostatic burden of the fish [27]. As the central organ for gas exchange and ion regulation in fish, the gills are a sensitive target of ammonia nitrogen toxicity. Sun et al. [28] observed that acute ammonia nitrogen stress can cause necrosis, desquamation, and vacuolization of gill epithelial cells in yellowfin tuna, as well as hyperplasia and fusion of secondary gill lamellae. Liu et al. [29] further reported that acute ammonia nitrogen stress was associated with gill lamellar fusion, epithelial necrosis, and inflammatory cell infiltration, accompanied by impaired gill tissue integrity and abnormal osmoregulation in yellow catfish. Although we did not directly examine gill tissue in the present study, these histological changes may impair oxygen uptake and metabolic waste excretion, requiring fish to increase their ventilation activity as a compensatory response. Therefore, the sustained increase in ventilation frequency observed in this study may indicate an increased ventilatory demand for maintaining gas exchange and internal homeostasis under acute ammonia nitrogen stress, but the underlying physiological mechanism remains inferential and requires further physiological validation. A similar compensatory ventilation response has also been observed in rice field eel under adverse water quality stress [3].

In contrast to the sustained increase in ventilation activity, changes in the locomotor behavior of rainbow trout were mainly characterized by reduced locomotor capacity and altered behavioral organization. Mondal et al. [30] found that zebrafish exhibited decreases in feeding and other normal behaviors, together with an increase in stress-induced locomotion, after continuous ammonia exposure. In this study, rainbow trout in the high ammonia nitrogen concentration group exhibited a decrease in average swimming speed, occasional short-term increases in activity, an overall reduction in activity level, and a shift in spatial distribution from relative aggregation to greater dispersion. This pattern may be partly explained by energy reallocation under stress. Yuan et al. [31] noted that fish under stress tend to prioritize resources for maintaining homeostasis and stress defense, whereas non-priority functions, including feeding, growth, and sustained activity, may be suppressed. Zhou et al. [32] further showed, through behavioral analysis and transcriptomic profiling of *Schizothorax prenanti*, that ammonia nitrogen stress was associated with changes in both behavior and energy metabolism-related pathways. Therefore, the decline in locomotor activity in rainbow trout may represent an adaptive adjustment that reduces energy expenditure and alleviates physiological burden. However, locomotor suppression cannot be fully explained by adaptive energy reallocation alone. He et al. [33] found that ammonia exposure reduced locomotor activity in zebrafish and was accompanied by increased ROS production, inflammatory responses, and glutamate/GABA imbalance. Zhang et al. [34] observed increased anxiety-like behavior in guppies under ammonia exposure. More recent evidence also suggests that ammonia nitrogen can directly affect fish brain tissue and induce neurotoxicity and oxidative damage [26]. These findings suggest that ammonia nitrogen stress may also impair fish locomotion by disrupting neural regulation. Accordingly, the more pronounced suppression of swimming ability in rainbow trout under high-concentration ammonia nitrogen treatment may not only reflect reduced locomotor activity, but also a deeper disruption of locomotor control and behavioral regulation.

It is worth noting that the ventilation activity and locomotor behavior of rainbow trout did not maintain their normal coordinated relationship under acute ammonia nitrogen stress. Under normal conditions, increased locomotor activity leads to higher metabolic oxygen demand, and ventilation frequency typically increases accordingly. In this study, ventilation frequency continued to increase, whereas average swimming speed and overall locomotor activity tended to decrease, indicating that the original coupling relationship between ventilation and locomotion was disrupted. Previous studies have suggested that different physiological and behavioral indicators may respond to stress on different time scales, and that a single indicator is often insufficient to fully represent fish state [35]. Therefore, ventilation frequency may reflect the immediate physiological compensation of rainbow trout under acute ammonia nitrogen stimulation, whereas changes in locomotor behavior more likely reflect subsequent functional suppression. Compared with a single variable, the combined analysis of multiple indicators can provide a more comprehensive interpretation of the response characteristics of rainbow trout under ammonia nitrogen stress.

Although this study revealed the differential responses of ventilation activity and locomotor behavior in rainbow trout under acute ammonia nitrogen stress, several limitations should be considered. The relatively small sample size may limit the statistical power and generalizability of the results, particularly for locomotor indicators that are susceptible to inter-individual variability. In addition, the analysis was limited to the video data from the final 5 min of each 1 h acute exposure recording. Although this standardized window helped reduce the influence of transfer- and environment-related disturbance, the use of a single time window may have limited the characterization of temporal changes in rainbow trout responses during acute exposure. The present experiment also lacked concurrent validation using physiological indicators, such as gill histopathology, blood biochemical parameters, and neurotransmitter levels. Future studies could build on this computer-vision-based framework by involving larger cohorts, incorporating multiple time-window analyses, extending the exposure duration and integrating histological, physiological, biochemical, and molecular analyses, thereby more systematically elucidating the response patterns of rainbow trout at different stages of ammonia nitrogen stress.

Despite these limitations, the proposed framework provides a potential basis for the non-invasive monitoring of behavioral and ventilatory changes in aquaculture systems. In commercial aquaculture settings, such as raceways, ponds, and recirculating aquaculture systems, the system could be implemented by installing fixed underwater or side-view cameras in representative monitoring areas and combining visual indicators with routine water-quality measurements. However, compared with the controlled laboratory conditions used in this study, commercial aquaculture environments are more complex. High stocking density, excessive water turbidity, strong surface reflection, and frequent inter-fish occlusion may reduce the stability of target detection, tracking, and mouth-region optical-flow analysis. Therefore, practical deployment would require further optimization of camera placement, illumination conditions, model robustness, and integration with water-quality sensors before large-scale application in aquaculture environments.

## 5. Conclusions

In this study, we developed a non-invasive vision-based framework to simultaneously quantify locomotor behavior and ventilation activity in rainbow trout under acute ammonia nitrogen stress. The results showed that ventilation frequency increased significantly with increasing TAN concentration, whereas locomotor activity was generally suppressed, as reflected by reduced average swimming speed and altered spatial distribution patterns. These divergent responses indicate that acute ammonia nitrogen stress induced distinct changes in ventilatory and locomotor behavior. Among the measured visual indicators, ventilation frequency showed a clearer and more stable concentration-dependent response than locomotor measures, suggesting that it may serve as a sensitive indicator for evaluating acute ammonia nitrogen stress in rainbow trout. This framework provides a methodological basis for non-invasive stress phenotyping and visual monitoring in aquaculture.

## Figures and Tables

**Table 1:** Dataset composition.

Dataset Split	Number of Images (3D Motion)	Proportion (%)	Number of Images (2D Ventilation)	Proportion (%)
Training Set	5740	70	3080	70
Validation Set	1640	20	880	20
Test Set	820	10	440	10
Total	8200	100	4400	100

**Table 2:** Performance comparison of fish-head and mouth-region detection models.

Task	Precision	Recall	mAP@0.5	mAP@0.5:0.95
Fish-head detection	0.986	0.963	0.986	0.695
Mouth-region detection	0.836	0.888	0.934	0.653

**Table 3:** Amount of exercise of rainbow trout under different ammonia nitrogen concentrations.

Ammonia Nitrogen Concentration (mg/L)	Amount of Exercise (cm)				
	Fish 1	Fish 2	Fish 3	Fish 4	Total
0	937.00	251.85	74.84	1319.17	2582.86
15	215.05	310.32	426.07	460.12	1411.56
30	448.56	266.84	473.59	592.12	1781.11
60	119.21	283.51	439.88	235.99	1078.59

## Data Availability

The authors do not have permission to share the data. The datasets generated and/or analyzed during the current study are not publicly available due to institutional restrictions but may be available from the corresponding author upon reasonable request.

## Abbreviations

The following abbreviations are used in this manuscript:

2D	Two-dimensional
3D	Three-dimensional
ANOVA	Analysis of variance
AP	Average precision
CPU	Central processing unit
DLT	Direct linear transformation
FN	False negative
FP	False positive
HSD	Honestly significant difference
IHNV	Infectious hematopoietic necrosis virus
IoU	Intersection over union
LED	Light-emitting diode
mAP	Mean average precision
NH_3_	Unionized ammonia
NH_4_^+^	Ionized ammonium
NH_4_Cl	Ammonium chloride
ONNX	Open Neural Network Exchange
PR	Precision–recall
RAS	Recirculating aquaculture system
ROI	Region of interest
TAN	Total ammonia nitrogen
TP	True positive
YOLO	You Only Look Once
